# Brain flexibility increases during the peri-ovulatory phase as compared to early follicular phase of the menstrual cycle

**DOI:** 10.1038/s41598-023-49588-y

**Published:** 2024-01-23

**Authors:** Marianna Liparoti, Lorenzo Cipriano, Emahnuel Troisi Lopez, Arianna Polverino, Roberta Minino, Laura Sarno, Giuseppe Sorrentino, Fabio Lucidi, Pierpaolo Sorrentino

**Affiliations:** 1 Department of Philosophical, Pedagogical and Quantitative-Economic Sciences, University of Chieti-Pescara “G. d’Annunzio”, 66100 Chieti, Italy; 2 Department of Motor Sciences and Wellness, University of Naples “Parthenope”, 80133 Naples, Italy; 3 Institute of Applied Sciences and Intelligent Systems, National Research Council, 80078 Pozzuoli, Italy; 4 Institute for Diagnosis and Cure Hermitage Capodimonte, 80131 Naples, Italy; 5 Department of Neurosciences, Reproductive Science and Dentistry, University of Naples “Federico II”, 80131 Naples, Italy; 6 Department of Social and Developmental Psychology, “Sapienza” University of Rome, 00185 Rome, Italy; 7 Institut de Neurosciences Des Systèmes, Aix-Marseille Université, 13005 Marseille, France; 8 Department of Biomedical Sciences, University of Sassari, 07100 Sassari, Italy

**Keywords:** Neuroscience, Dynamical systems, Network models

## Abstract

The brain operates in a flexible dynamic regime, generating complex patterns of activity (i.e. neuronal avalanches). This study aimed at describing how brain dynamics change according to menstrual cycle (MC) phases. Brain activation patterns were estimated from resting-state magnetoencephalography (MEG) scans, acquired from women at early follicular (T1), peri-ovulatory (T2) and mid-luteal (T3) phases of the MC. We investigated the functional repertoire (number of brain configurations based on fast high-amplitude bursts of the brain signals) and the region-specific influence on large-scale dynamics across the MC. Finally, we assessed the relationship between sex hormones and changes in brain dynamics. A significantly larger number of visited configurations in T2 as compared to T1 was specifically observed in the beta frequency band. No relationship between changes in brain dynamics and sex hormones was evident. Finally, we showed that the left posterior cingulate gyrus and the right insula were recruited more often in the functional repertoire during T2 as compared to T1, while the right pallidum was more often part of the functional repertoires during T1 as compared to T2. In summary, we showed hormone-independent increased flexibility of the brain dynamics during the ovulatory phase. Moreover, we demonstrated that several specific brain regions play a key role in determining this change.

## Introduction

Human brain architecture enables efficient information processing, supporting complex cognitive and behavioral functions^1^. Such complexity is underpinned by locally and globally integrated hierarchical and modular dynamics^2^, with simple components operating at different, nested spatio-temporal scales.

Network theory lends itself to explaining the functioning of a complex and integrated system such as the human brain^3^. Accordingly, the brain has been conceptualized as a graph whose nodes are the anatomical regions, and whose edges are the connections (either structural or functional) among them. Within this framework, structural and functional connectivity studies have measured anatomical connections (structural connectivity) and statistical dependencies (functional connectivity) between the activities of pairs of brain regions^1^, both in physiological and pathological conditions. However, most of the functional metrics used to this date, focused on the average connectivity over a time interval, assuming stationareity of the brain, thus disregarding the fast, multimodal evolution of the coactivations over time (see Table 1 for a comprehensive explanation)^4–6^. This shift in perspective from a 'static' to a 'dynamic' point of view has allowed for the exploration of brain activities with new methodological approaches and new metrics to measure and characterize time-varying functional connections. Converging evidence demonstrates that brain dynamics is pivotal to neural and behavioral adaptability.

**Table 1:** Glossary of technical terms.

Concept	Description	Reference
Brain dynamic	It explores the temporal evolution of brain activity, including the transmission of information between brain regions and the variation of their statistical dependencies, which might underpin to complex behaviour	Zalesky et al. (2014)^4^
Criticality	Critical systems exhibit scale-free fluctuations, and produce a dynamics which is “between order and disorder”. Critical systems can typically be explored by robust statistical analysis and modelling. A critical system operates in a highly variable, adaptive, and flexible dynamic regime	Cocchi et al. (2017)^7^
Brain flexibility	It refers to the brain's ability to explore a large number of its possible dynamical states	Pinto et al. (2022)^8^
Neuronal avalanches	A cascade of neural events (e.g. action potentials) that starts from a single event and propagates throughout the network, typically across spatial and temporal scales These cascades have been described by a critical branching process	Beggs et al. (2003)^9^
Branching ratio (σ)	The ratio between the number of activations in consecutive time steps. When σ = 1, the system is operating at criticality	Haldeman et al. (2005)^10^
Functional Repertoire	The number of unique avalanche configurations (pattern) that occurred during the recording. The size of the functional repertoire represents a measure of flexibility of brain activity. For a graphical representation see Fig. 2	Ribeiro et al. (2016)^11^
Shared functional repertoire	Avalanche pattern that occurs in more than one phase of the menstrual cycle. For a graphical representation see Fig. 2	Sorrentino et al. (2021)^12^
Phase specific functional repertoire	Avalanche pattern specific to only one phase of the menstrual cycle. For a graphical representation see Fig. 2	Sorrentino et al. (2021)^12^
Switches	Occurs when the value of a time series crosses the threshold in either direction (an active region becomes inactive, and vice versa)	Sorrentino et al. (2021)^12^

An approach that takes into account dynamical variations in signal amplitude across all brain signals, defined as *neuronal avalanches* (for a full explanation see Table 1), has been proposed^9,12^.

Studies on emergent properties of dynamic systems^13,14^ might suggest that the brain operates in or near a critical regime (for a full explanation of the concept of criticality, see Table 1) that optimizes information transmission and processing*.* Accordingly, brain activities generated in the human brain are typically not stereotyped but, rather, they give raise to large, non-linear bursts of activities that constantly reconfigure themselves in space and time^9,14–16^.

In this manuscript, we operationally defined a neuronal avalanche, as an event that begins when the amplitude of at least one region deviates from its baseline and ends when all regions restore their typical amplitude. Previous studies analysed the high-amplitude burst activity of the brain through the neuronal avalanche approach in resting state conditions, demonstrating power-law probability distributions of the size and duration of the avalanche and showing a balance between ongoing and future neuronal activity^17–19^. An *avalanche pattern* is the set of all the brain areas that were recruited during an avalanche, and the *functional repertoire* is the set of the unique avalanche patterns (for a full explanation see Table 1). This means that any pattern that repeats itself over time only counts one towards the definition of the functional repertoire (i.e., the repetitions are discarded). The functional repertoire measure brain flexibility, defined as the ability to dynamically recruit different subset of regions over time. Evidence shows that an "optimally" flexible state (i.e., with an optimal trade-off between stereotyped and random dynamics) is the most efficient configuration for the resting brain dynamics^20^.

The menstrual cycle (MC) is a process originating from the coordinated variation of different sex hormones^18,19^, aimed at the reproductive process. However, the MC affects the body well beyond the reproductive organs, also inducing changes in the functioning of the central nervous system and in the women's emotional and behavioural states. For this reason, neuroadaptive mechanisms that modulate brain structure and function during the MC have been hypothesized, as might be suggested by structural changes in the hippocampus, amygdala as well as in the temporal and parietal regions^21–23^. Furthermore, changes have been demonstrated also in brain connectivity, where the brain topology has been related to the phase of the MC and to the corresponding hormonal changes^24–28^ associated with clinically relevant conditions, such as the premenstrual syndrome or the premenstrual dysphoric disorder^29,30^. Moreover, a previous study based on quantitative parameters derived from magnetoencephalography (MEG), such as median and peak alpha frequency of the power spectrum and Shannon spectral entropy, showed that spontaneous neural oscillations change during the MC in the delta, theta, and gamma frequency bands^31^. Despite the evidence that brain structural and functional change associate with the MC, to our knowledge, few studies have focused on the changes of the brain dynamics along the MC. In particular, De Filippi et al.^32^, used a measure of turbulence to demonstrate changes in the whole-brain dynamics linked to ovarian hormones and menstrual cycle phases. Furthermore, Mueller et al.^33^, through dynamic community detection techniques, demonstrated that hormonal changes during the menstrual cycle result in temporary and localized patterns of brain network interactions. However, these two studies are based on a densely sampled data set from a single participant. In this manuscript, we use a larger sample of participants, although with a lower sampling rate over time (ie. three time points, as explained), to clarify the role of the menstrual cycle on brain dynamics.

In the present study, we hypothesized that the brain dynamics, explored through the prism of neuronal avalanche, cyclically change along the MC. To verify our hypothesis, we evaluated the functional repertoire at three time points of the MC (i.e. early follicular, peri-ovulatory and mid-luteal phases). Moreover, our study aimed at verifying the hypothesis that possible modifications in brain dynamics may be linked to changes in blood levels of sex hormones.

To test our hypothesis, we used a magnetoencephalography (MEG) system to measure the brain dynamic patterns of activation in resting state, in healthy naturally-cycling women without premenstrual symptoms and with no signs of anxiety and/or depression. In particular, we applied the *''neuronal avalanches''* framework to describe the variability of the brain functional repertoire along the MC. Furthermore, during the three phases of the MC, blood samples were collected to determine hormone levels of estradiol, progesterone, luteinizing hormone (LH), and follicular-stimulating hormone (FSH) in order to test the hypothesis of a correlation between changes in brain dynamics and sexual hormones. Finally, we analyzed the occurrence of brain regions in specific and shared functional repertoires.

## Methods

### Participants

For our study, twenty-seven females were recruited (age and education 26.6 ± 5.1 and 17.3 ± 2.7 years, respectively). They are native Italian speakers, right-handed and heterosexual with a regular MC. At enrolment, all women signed informed consent. All the procedures were conducted in accordance with the Declaration of Helsinki, IV edition. The study was approved by the Local Ethics Committee of University of Naples “Federico II” (protocol n. 223/20). We selected women with regular MC (mean cycle length 28.4 ± 1.3 days), who did not use drugs or medicines, including the hormonal contraceptives (or other hormone regulating medicament) during the last six months before the start of the study, since these drugs might affect both the central nervous system and the MC. We excluded participants who became pregnant in the last year, with a history of neuropsychiatric diseases or premenstrual dysphoric/depressive symptoms. Furthermore, the participants did not consume tobacco, alcohol and coffee in the 48 h preceding the MEG recordings. To control for the influence of circadian rhythm, the time of testing varied no more than two hours between testing sessions. The Beck Depression Inventory (BDI)^34^ and Beck Anxiety Inventory (BAI) ^35^ were used to test the mood and/or anxiety symptoms. Based on the results of these two tests, two females were excluded from the study as they exceeded the cut-off (10 and 21, respectively).

### Experimental protocol

The women were acquired in three different time points of MC, at early follicular (cycle day 1–4, low estradiol and progesterone, T1), during the peri-ovulatory (cycle day 13–15, high estradiol, T2), and in mid-luteal (cycle day 21–23, high estradiol and progesterone, T3) phases. We applied the back-counting method to define the individual time points cycle. The self-reported onset of menses was used as a starting point to estimate in an indirect way the peri-ovulatory and mid-luteal windows. At each time point of the cycle, we performed the MEG recordings and the blood sample collection for the analysis of the sex hormones, namely estradiol, progesterone, FSH, and LH. During the early follicular phase, we also carried out a transvaginal pelvic ultrasonography examination. Finally, after the last MEG recording, structural magnetic resonance imaging (MRI) was performed. To control for a possible session effect, we randomised the MC phase from which the three recording sessions started. For a full explanation of the procedures for the analysis of hormone levels, the pelvic ultrasound, and the MRI, see supplementary information (SI).

### Brain dynamics analysis

**Figure 1:**
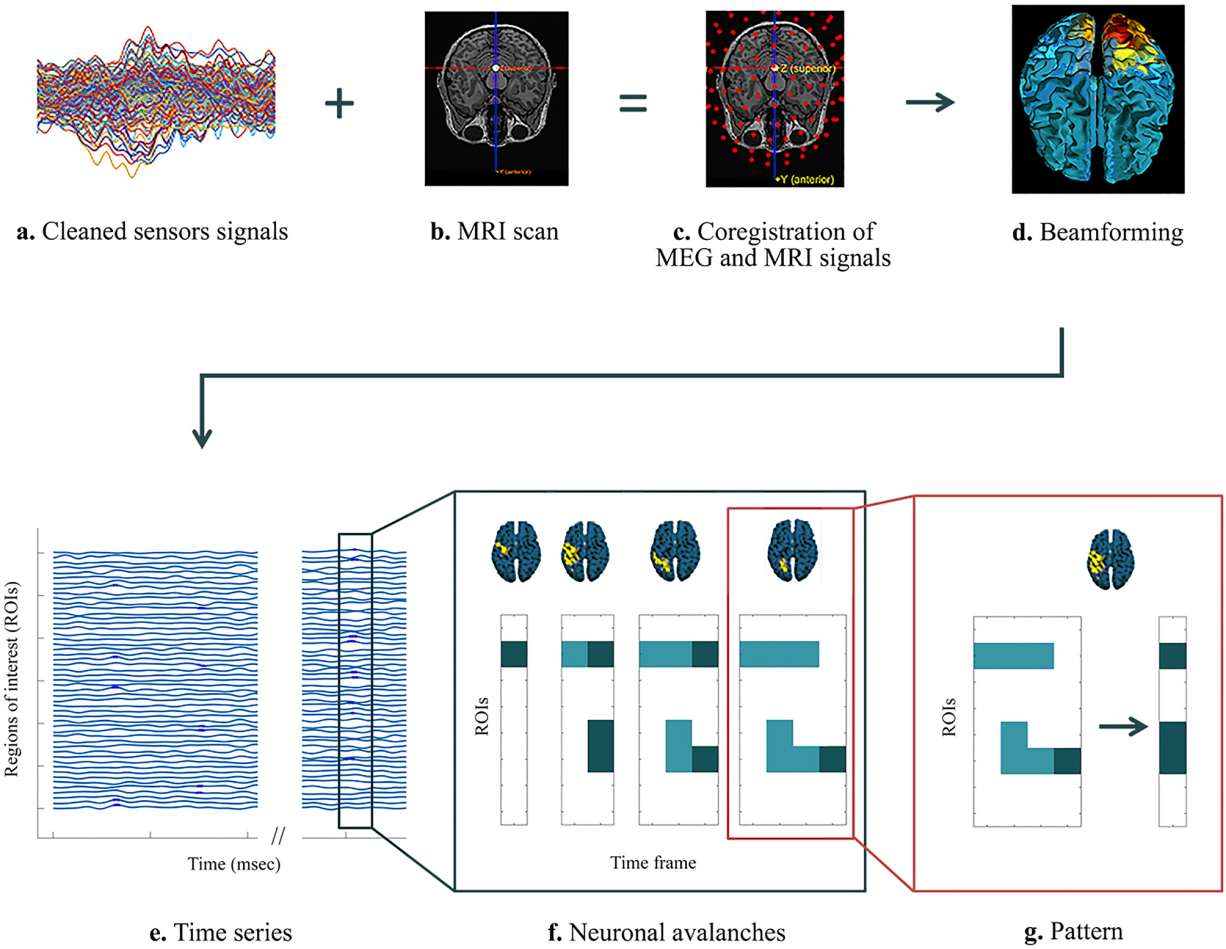
Data analysis pipeline and Neuronal Avalanches representation. (**a**) Magnetoencephalography (MEG) sensor-level signals after preprocessing and cleaning. (**b**) Structural magnetic resonance imaging (MRI). (**c**) Structural MRI, MEG sensors and the position of the participant's head are co-registered in the same coordinate system. (**d**) Through a Beamformer algorithm, the time series of the sources are estimated in regions of interest within the brain according to a parcellation based on the ALL atlas. (**e**) In green, source-reconstructed time series of regions of interest. The dark green rectangle highlights the time interval in which a neuronal avalanche occurs. An avalanche is defined as an event that begins when the amplitude of at least one brain region exceeds the threshold value—equal to three standard deviations (z =  ± 3) away from its baseline amplitude—and ends when all regions have their amplitude below the threshold. The blue dots included in the rectangle define the brain regions whose amplitude is above the threshold value in a given time interval (msec). (**f**) Frame by frame development of neuronal avalanches. Above, the set of all brain areas recruited during the avalanche (in yellow all the areas whose amplitude is above threshold and in green the areas whose amplitude is below threshold). At the bottom, the evolution in time of the avalanche is represented as follows: in each box, the dark green squares indicate the brain regions (ROIs) activated in a specific time frame, while the light green indicates the regions that were active in the previous frames. (**g**) Starting from the avalanche (to the left), we can calculate the patterns of activation (to the right), taking into consideration all the brain regions that were active during the avalanche, as represented in the brain plot above. The number of unique avalanche patterns defines the size of the functional repertoire and it is used as a proxy to estimate the flexibility of brain dynamics. The figure was made using Matlab 2019a.

Brain magnetic signals were recorded through a MEG system, equipped with 163 magnetometers (154 channels and 9 references), based on superconducting quantum interference devices (SQUIDs) and placed in a shielded room (AtB Biomag UG, Ulm, Germany)^36^. This system is characterized by a magnetic field noise spectral density of approximately 5 fT/Hz1/2 (For an extensive explanation of the characteristics of the system, please refer to the study by Rombetto et al.^37^). Before registration, the head position was identified, digitizing the coordinates of four anatomical landmarks and four position coils^38^. Each participant underwent two eyes-closed recordings lasting 3′30’’ each. Between the recordings, the participants took a short break during which they remained in the MEG room, could open their eyes, and stretch their legs and arms while remaining seated. Electrocardiogram and electro-oculogram were also acquired, to identify physiological artefacts^39^. The brain signals were then cleaned through an automated processes as reported in our previous article (Fig. 1a)^40^. The FieldTrip software tool^41^, based on Mathworks® MATLAB, was used to implement principal component analysis (PCA), to reduce the environmental noise. To remove physiological artefact, such as cardiac noise or eye blinking (if present), independent component analysis (ICA) was used. For each participant, source reconstruction was performed through a beamforming procedure (Fig. 1d) utilizing the Fieldtrip toolbox, similar to Pesoli et al.^42^. In short, based on the native MRI, the volume conduction model proposed by Nolte^43^ was applied, and the Linearity Constrained Minimum Variance^44^ beamformer was implemented to reconstruct the time series related to the centroids of 116 regions-of-interest (ROIs), derived from the Automated Anatomical Labelling (AAL) atlas (Fig. 1b,c). We only considered the first 90 ROIs, excluding those corresponding to the cerebellum, given that the reconstructed signal might be less reliable^45^. The source-reconstructed signals were filtered in the classic frequency bands: delta (0.5–4.0 Hz), theta (4.0–8.0 Hz), alpha (8.0–13.0 Hz), beta (13.0–30.0 Hz), and gamma (30.0–48.0 Hz). Broadband data were also analyzed^46^. Furthermore, we also tested the subject-specific alpha band, by adjusting the cut-off frequencies according to a window of ± 3 Hz around the individuals’ alpha peak.

We started from the time series of 90 ROIs (Fig. 1e) to estimate the *Neuronal Avalanches*^14^, defined as an event during which the amplitude of the signals exceeds a certain established threshold value (Fig. 1f). This event begins when at least one region becomes active (i.e., above threshold) and continues as long as any region remains above the threshold, and can involve both adjacent areas and areas of different hemispheres. The combination of all the brain regions that were involved in a specific avalanche is defined as “avalanche configuration” (or avalanche pattern) (Fig. 1g). Note that, to reduce data dimensionality and simplify the analyses, time is collapsed within each avalanche. Before estimating the fluctuation of the signal’s amplitude, we standardized each signal by calculating its z-score and establishing a threshold value equal to three standard deviations (z =  ± 3)^47^. Hence, a region was considered as “active” only when its score was above threshold. However, to demonstrate that the results are not strictly dependent on the chosen threshold, we repeated the analyses by setting two other cut-offs equal to + 10% and − 10% of the initial threshold value (z = 3.3 and z = 2.7).

Dynamic analysis of neuronal signals requires the time series to be binned, to ensure one captures a critical phenomenon, if present. To select a suitable bin length, we computed the branching ratio σ^10,48^. In other words, for each time bin duration, for each subject, for each avalanche, the (geometrically) averaged ratio of the number of events (activations) between the subsequent time bin and that in the current time bin was calculated as:

1 $$\sigma_{i} = \mathop \prod \limits_{j = 1}^{{N_{bin} - 1}} \left( {\frac{{n_{events} \left( {j + 1} \right)}}{{n_{events} \left( j \right)}}} \right)^{{\frac{1}{{N_{bin} - 1}}}}$$

where σ_i_ is the branching parameter of the i-th avalanche in the dataset, Nbin is the total number of bins in the i-th avalanche, n events j is the total number of events in the j-th bin. Subsequently, we (geometrically) averaged the results over all avalanches as:

2 $$\sigma_{i} = \mathop \prod \limits_{i = 1}^{{N_{aval} }} \left( {\sigma_{i} } \right)^{{\frac{1}{{N_{aval} }}}}$$

where N*aval* is the total number of avalanches in each participant’s dataset. In critical processes, a branching ratio ~ 1 indicates critical processes, σ < 1 indicates subcritical processes in which the activity quickly dies out, and σ > 1 indicates supercritical processes with runaway excitations. We averaged the branching ratio across all participants, in all time points, for each bin size, to even out the results and make the calculated parameters comparable. While the branching ratio was close to one for all the bins that we tested, and the statistical results were confirmed for all bins, we report in the manuscript the results for bin = 4, since this was the closest to 1.

**Figure 2:**
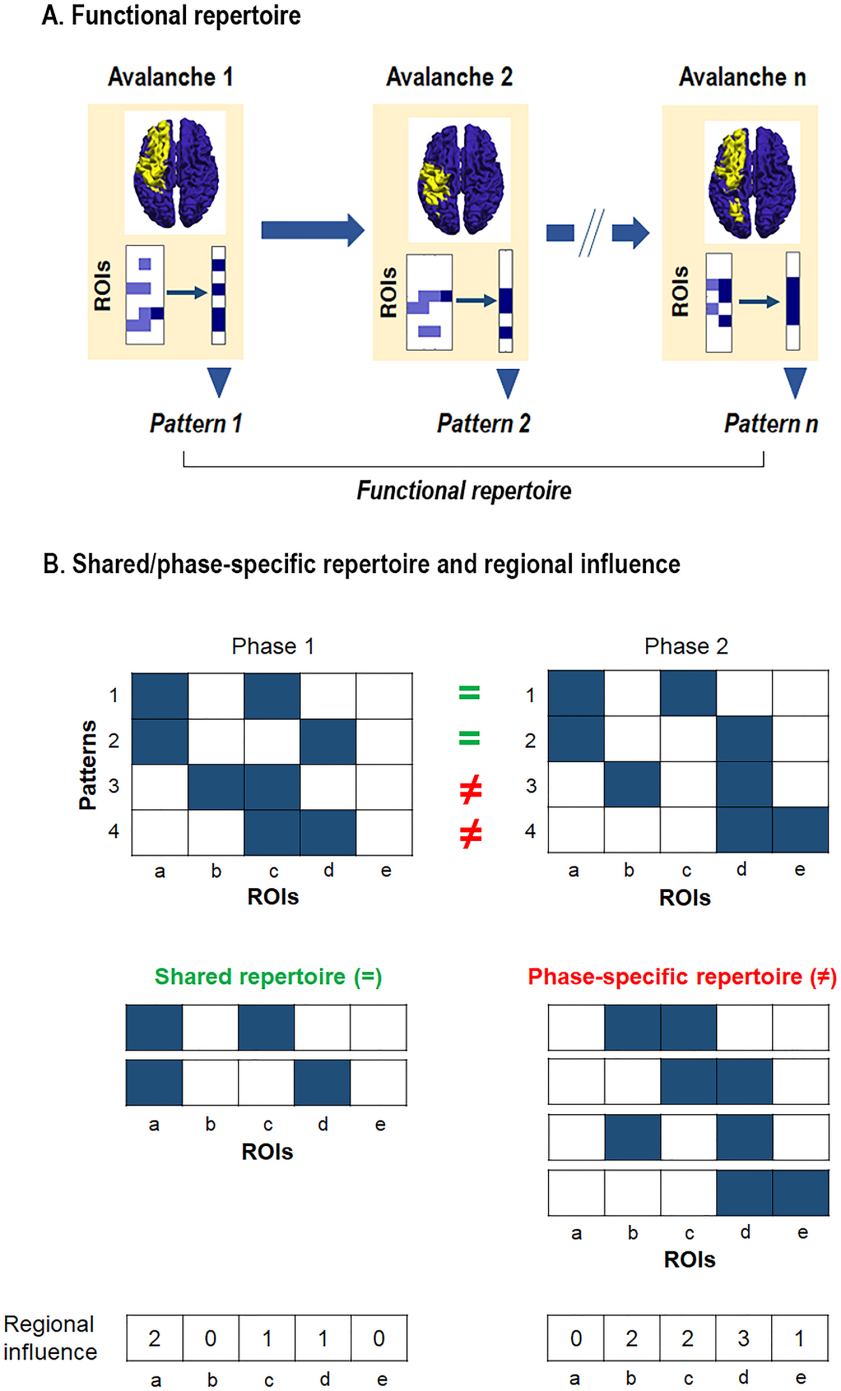
Graphical representation of functional repertoire, shared/phase-specific repertoire and brain regional influence. (**A**) Schematic representation of the functional repertoire. Specifically, in the boxes, for each avalanche (i.e. avalanche 1, avalanche 2, …, avalanche n), the matrix represents an avalanche pattern: dark blue squares indicate the brain regions (ROIs) activated at a certain time frame, while the light blue ones are all the regions that have been activated up to that moment. The transition from activation to inactivation, and vice versa, between two consecutive time frames is defined as switch. Brain-plots and the set of unique avalanche patterns (i.e. pattern 1, pattern 2, …, pattern n) are illustrated for each avalanche. The number of unique avalanche patterns defines the size of the functional repertoire and is used as a proxy for the flexibility of the brain dynamics. (**B**) Schematic representation of shared and phase-specific repertoires. Blue squares depict the brain regions whose activity is over threshold during the avalanche (unique avalanche patterns); phase 1 and phase 2 share patterns 1 and 2 (green “ = ” symbol), while patterns 3 and 4 are phase-specific (red “ ≠ ” symbol). Regional influence is given by the sum of each region's activations. Specifically, region “a” is only involved in the shared repertoire (2 times), while regions “b” and “e” are solely involved in the phase-specific repertoire (twice and ones, respectively). The figure was made using Matlab 2019a.

For the dynamic evaluation of neuronal activity, we estimated the following information: the *functional repertoire* composed by the number of unique avalanche configurations that was expressed during the recording; the *switches* between each region state (i.e. above—below threshold), represented by a crossing of the threshold in either direction between two consecutive time–bins; and the *regional influence on neuronal avalanches pattern*, which allows to estimate the role of each region within the MC phase-specific functional repertoires. Specifically, to estimate the regional influence on the neuronal avalanche patterns, the functional repertoire has been divided into two components: the patterns that occurred in different phases of the MC, namely the “*shared repertoire*” and the patterns that characterized the functional repertoire of one specific phase of the MC, defined as “*phase-specific repertoire*”. Subsequently, we used the Kolmogorov–Smirnov test to compare the distribution of the frequencies between the two repertoires across all regions. After the distributions were demonstrated to be statistically different, the regions were compared, individually, as an uncorrected post-hoc analysis. Finally, we performed a permutation test to identify which brain regions occurred significantly more and in which repertoire (i.e., specific or shared). A brief description of the pipeline used and a schematic representation of neuronal avalanches are shown in Fig. 1. Finally, a glossary of technical terms is reported in Table 1, and a graphical representation of the functional repertoire, shared/phase-specific repertoires, and brain regional influence are reported in Fig. 2.

### Statistics

Statistical analysis was carried out using MATLAB (Mathworks®, version R2013a). We checked the normal distribution of variables using the Shapiro–Wilk test. To compare, in all frequency bands and all bins, the neuronal avalanches data among the three time points of the MC, we used the Friedman test. All the p values were corrected for multiple comparisons using the false discovery rate (FDR)^49^. Subsequently, the post hoc analysis was carried out using the Wilcoxon signed rank test. The relationship between hormone blood levels and dynamic brain features was investigated through Spearman's correlation test and multilinear regression analysis, using either the values of the specific phases, the values of the transition between two phases (Δ), or the overall variation of the hormones as assessed using the magnitude of the first component resulting from the PCA decomposition (see SI for more details). The statistical significance was defined as *p* < 0.05 after FDR correction.

### Ethical approval

All procedures performed were in accordance with the ethical standards of the institutional research committee and with the ethical standards laid down in the 1964 Declaration of Helsinki and its later amendments.

### Informed consent and consent to participate

Written informed consent has been obtained from all participants.

## Results

### Brain dynamic analysis estimated by functional repertoire and switch rate

**Figure 3:**
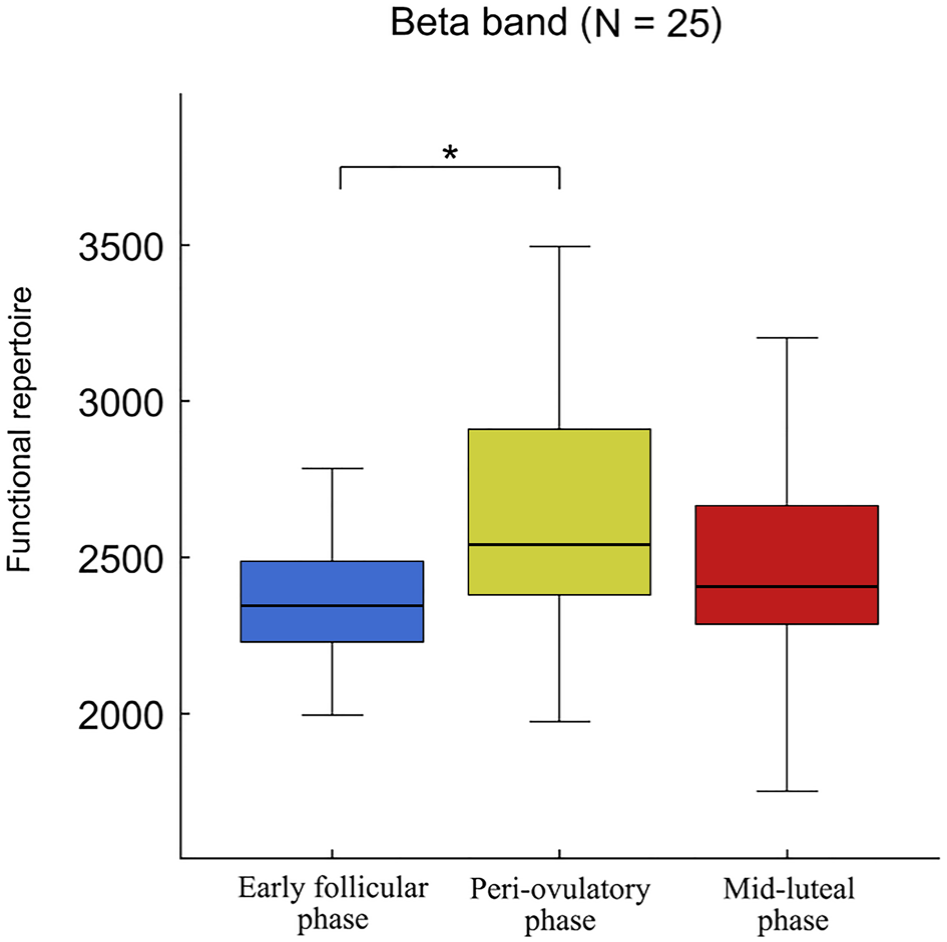
Neuronal Avalanches comparison. The box plots refer to the comparison of functional repertoires (the total number of unique avalanche patterns that occurred in each MC phase) in the beta band, estimated in 25 women (N = 25) during the MC. In each box plot, the values are shown at early follicular, peri-ovulatory and mid-luteal phases. The upper and lower bound of the rectangles refer to the 25th to 75th percentiles, respectively. The median value is represented by a horizontal line inside each box, the whiskers extend to the 10th and 90th percentiles. The box plots show a significantly higher functional repertoire during the peri-ovulatory phase, as compared to the early follicular (*p* = 0.026) phase. Significance *p* value: *p < 0.05. The figure was made using Matlab 2019a.

We measured the branching ratio of the time series binned with different lengths in order to observe the system in its critical state. For a threshold of z = 3, bin 4 showed a branching ratio σ = 1, hence our analyses were performed using time series in which each bin was obtained including four time points. The statistical comparison of the size of the functional repertoire across the three phases, based on the Friedman test, showed a significant difference among the functional repertoire of the three phases (χ^2^ (df = 2, N = 25) = 8.24, *p* = 0.016, pFDR = 0.048), in the beta frequency band. In detail, the post-hoc analysis showed significantly more visited configurations during the peri-ovulatory phase, as compared to the early follicular (*p* = 0.026) phase (Fig. 3). To prove the robustness of our results with respect to both the avalanche threshold and the bin length, we tested these variables using different bins (1 to 5) and different thresholds (z = 2.7; z = 3; z = 3.3). These analyses revealed that the branching ratio remains very close to one for all the bins that were explored. Furthermore, the statistical results we obtained are stable across bins (bin t = 1, *p* = 0.023; bin t = 2, *p* = 0.026; bin t = 3, *p* = 0.026; bin t = 5, *p* = 0.026). Finally, the results were confirmed when testing different avalanche thresholds (*p* = 0.016 for z = 3.3, and *p* = 0.039 for z = 2.7).

No significant differences in the size of the functional repertoire were found in the remaining frequency bands. With respect to the analyses of the switches, no statistically significant differences were found in any frequency band (nor in broadband). The results are reported in the SI. Furthermore, for both the functional repertoire and the switches, no significant results were found when investigating the individually adjusted frequency range, calculating the alpha frequency windows individually.

### Relationship between brain dynamics and blood sex hormones levels

Using a multilinear model, Spearman correlations, and principal component analyses, we investigated whether the changes in the size of the functional repertoire could be mediated by changes in sex hormone levels during the MC. In particular, the correlations were investigated at each time point. All these analyses failed to demonstrate a relationship between variation in activation patterns and hormonal fluctuations. See SI for more details.

### Regional influence on neuronal avalanches pattern

**Figure 4:**
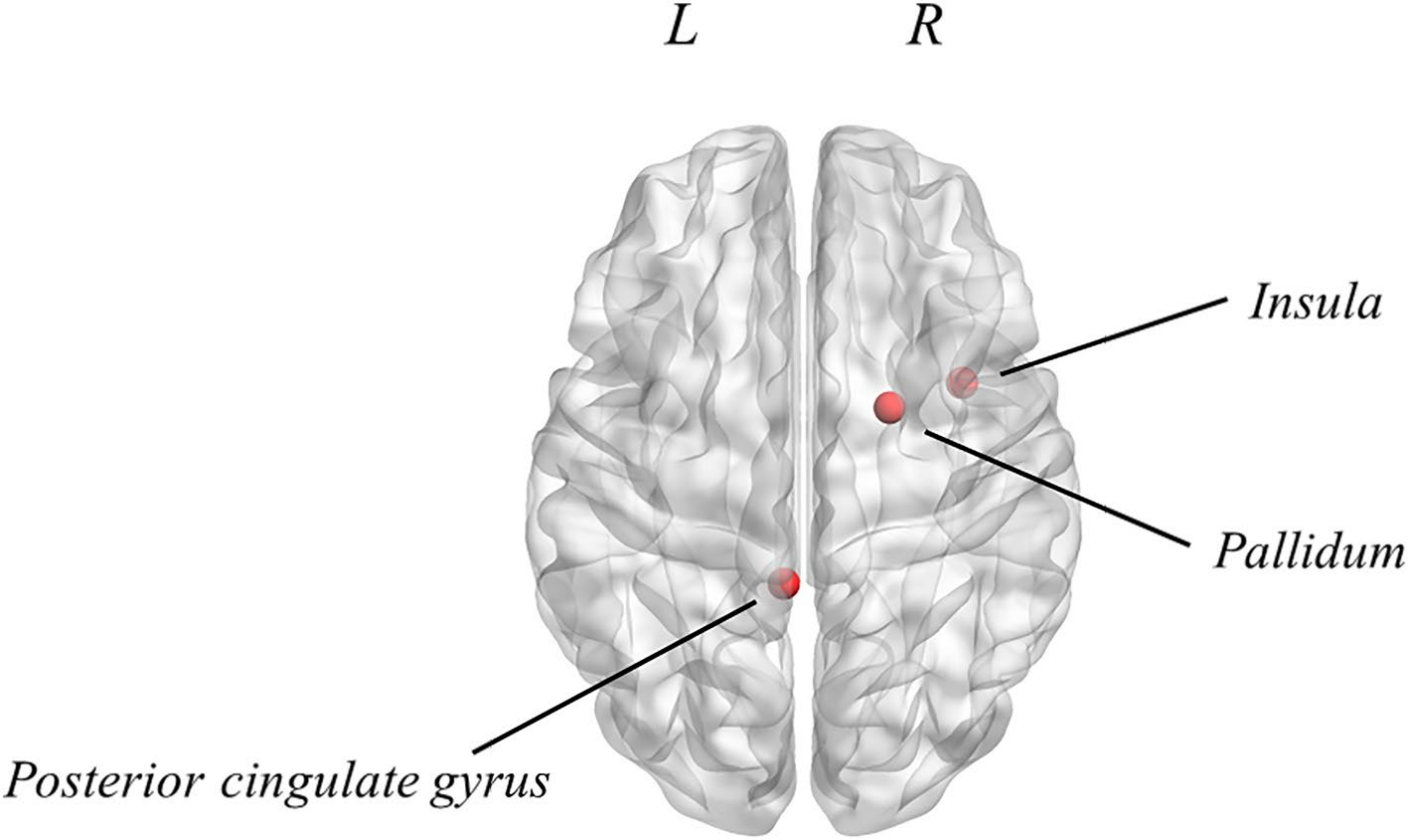
Mapping of brain regions occurring significantly more in the phase-specific unique avalanche patterns during the MC. In the beta frequency band, the left posterior cingulate gyrus (PCG) (*p* = 0.047) and the right insula (*p* = 0.033) occur more in the phase-specific repertoire of the peri-ovulatory phase than that of the early follicular phase. Furthermore, in the same frequency band, the right pallidum (*p* = 0.021) is recruited more often in the phase-specific repertoire of the early follicular phase, as compared to the one of the peri-ovulatory phase. The image was made using MatLab 2019a, including BraiNetViewer v. 1.62. Abbreviation: L (Left hemisphere); R (Right hemisphere).

With respect to the recruitment of specific brain regions in the phase-specific functional repertoire, we firstly compared the occurrence of specific regions in the shared repertoire (i.e. the patterns that were present irrespective of the phase of the MC) with respect to the phase-specific repertoires. For this analysis, we focused on the phase-specific repertoires of those phases that demonstrated a statistically different number of patterns (i.e., early follicular and peri-ovulatory phases). The Kolmogorov–Smirnov test revealed a statistically significant difference in the distribution of occurrences of the brain regions between the shared and the phase-specific repertoires of MC (*p* < 0.001) (Fig. 4). Subsequently, we identified which brain regions occurred significantly more in the phase-specific repertoire than in the shared one, by performing a post-hoc analysis based on a permutation test. The results highlighted that, in the beta frequency band, the left posterior cingulate gyrus (PCG) (*p* = 0.047) and the right insula (*p* = 0.033) are recruited more often in the functional repertoire of the peri-ovulatory phase, as compared to the one of the early follicular phase. Furthermore, in the same frequency band, the right pallidum (*p* = 0.021) is recruited more within the repertoire of the early follicular phase than within that of the peri-ovulatory phase.

## Discussion

In the present study, we set out to test the hypothesis that brain dynamics varies across the MC, and we tested if those changes are linked to physiological fluctuations of sex hormones that take place during the MC.

A robust literature suggests that the brain, in physiological conditions, changes its configuration across multiple temporal and spatial scales. The high number of configurations that are generated has been linked to the ability to adapt to a changing environment^2,3^. Here, we start from the evidence that the brain network topology, as observed in MEG using static connectivity metrics, undergoes modifications across the MC, as demonstrated in our previous study^50^. In the present work, we aimed to investigate whether the MC affects the dynamical properties of the brain networks. We applied a recently implemented technique based on the concept of “*avalanche*^9^”, through which we studied the variation of the size of the *functional repertoire* during the MC as a proxy of the brain's flexibility. While avalanches are typically related to the presence of a dynamics operating near a phase transition, our work does not aim at making a statement about the underlying dynamical regime. Rather, an operational definition of avalanches is adopted as a way to provide a readout of the flexibility of the dynamics.

Our results showed that the neuronal avalanches undergo profound rearrangements in different moments of the MC, as highlighted by the larger functional repertoire observed during the peri-ovulatory phase, as compared to the early follicular phase, in the beta frequency band. We speculate that, during the fertility period, it can be especially advantageous for women to optimally adapt to a variety of stimuli. Hence, one would expect the brain dynamics to be maximally flexible and, hence, the functional repertoire to be larger^51–53^.

Our result is in accordance with the results of Muller et al. who observed a temporary dynamic reorganization of the brain networks during the ovulatory phase. Starting from these changes, we tested whether we could demonstrate an association between sex hormones (in particular, estradiol, progesterone, FSH, and LH) and the changes in the size of the functional repertoire that observed during the MC. To this aim, we first build multilinear models to predict the number of patterns of the functional repertoire in T1, T2 and T3, as well as the observed changes between the two phases in Δ(T2 − T1), from the hormonal levels. However, none of the models could significantly predict the functional repertoire (see Supplementary Figures S1, S2, S3 and S4). Subsequently, we explored the relationship between the number of unique avalanches and sex hormones, both individually and by extracting the first principal component of the hormones variations, using principal component analysis. The analysis was repeated again for T1, T2 and Δ(T2 − T1). As reported in SI, the analysis failed to demonstrate the association between sex hormones and the number of unique avalanche patterns. In summary, the analyses that we carried out did not demonstrate a linear relationship between the sex hormones and the increase of functional repertoire observed during the peri-ovulatory phase. Although there is evidence^32,33^ that demonstrates an association between changes in estradiol and progesterone concentrations and the whole-brain dynamical reorganization on the large scale, this was not replicated in the current study. It should be noted that the previous evidence is based on a densely sampled dataset obtained from a single participant. However, it must be considered that the regulation of the menstrual cycle depends on multiple different factors. For instance, the release of FSH and LH is induced by the gonadotropin-releasing hormone, secreted by the hypothalamus, whose regulation, in turn, depends on many factors determined by proteins, cytokines, and other physiological processes ^54^. Furthermore, it is important to note that menopausal symptoms are also caused by fluctuating levels of sex hormones, but their levels do not explain the severity of symptoms. This might explain the lack of correlation between brain parameters and hormone levels across the MC. Hence, growth of the functional repertoire during the peri-ovulatory phase may be the outcome of more complex mechanisms, involving broader physiological processes. Further analysis, considering a larger panel of molecular pathways, could clarify this issue.

We also analyzed the number of switches that occurred, as a way to account for the possibility that the dynamics would not change qualitatively at the large scale but, rather, that the smaller functional repertoire would be derived from impaired local dynamics. However, we could not find any difference in terms of switch rates across the MC, demonstrating that the local dynamics are unchanged, despite the fact that the number of visited states (i.e. combinations of active regions defined at the large-scale) changes as a function of the phase of the MC. After observing the size of the functional repertoire, we investigated whether some brain regions recurred more than others in the functional repertoire, and we observed that several brain regions were recruited more often in phase-specific functional repertoires. In particular, our results showed the involvement of several areas of the limbic system including the PCG, the insula and the basal ganglia (pallidum). A previous MEG study^31^ that investigated regional neural oscillations in the resting state, showed that theta intensity in the right temporal cortex and the right limbic system was significantly lower during the menstrual period than outside the menstrual period. While this study used a different methodology and showed the involvement of different parts of the limbic system, it confirms that the involvement of the limbic system may be dependent on the menstrual cycle. It is interesting to note that the limbic system is a complex system, whose relevance for controlling the emotional experience is well established^55^.

In details, the PCG might play a role in the regulation of brain dynamics during the peri-ovulatory phase. From a functional point of view, according to Leech and colleagues, the relevance of the PCG in the functional repertoire during the peri-ovulatory phase could depend on the role of this area in the assessment of the decision's outcome^56^.

Another area of the limbic system that seems to have greater relevance in the pattern of unique avalanches during the peri-ovulatory phase is the right insula. Although the insula is a small brain region, it is connected, at the large scale level, with several neuronal circuits involved in the regulation of visceral and somatic stimuli, in socio-emotional modulation, and in cognitive processes^57^. It was observed that its dysfunction has been associated with the subjective experience of pain during the menstrual cycle^58^. One might speculate that the occurrence of this area in the functional repertoire of the peri-ovulatory phase could depend on the fact that the insula contributes to brain dynamics more prominently in the peri-ovulatory phase, perhaps favouring the expression of prosocial behaviours. However, this interpretation is purely speculative at this stage.

Finally, our study highlighted changes in the involvement of the basal ganglia (pallidum) in the functional repertoire throughout the menstrual cycle. Specifically, the right pallidum is recruited more often during the early follicular phase with respect to the peri-ovulatory phase. This result highlights the role of the basal ganglia in shaping the dynamics and functional repertoire of the brain. The involvement of the basal ganglia during the menstrual cycle has been observed in previous studies, although these did not investigate brain dynamics. In particular, the basal ganglia appear to be involved in the inhibitory control processes, which is the ability to modulate (suppress or stimulate) cognitive processes in order to select one action over another^59^. In this perspective, the basal ganglia seem to modulate the expression of impulsive behaviour.

The results of our study are specific in the beta frequency band. In line with our results, a study by Becker et al.^60^, based on EEGs during the menstrual cycle, showed that the power of the beta bands was also significantly higher in the phases when the blood level of progesterone was low (and in case of low temperature). However, fMRI, EEG, and MEG studies during the menstrual cycle also demonstrated the involvement of other frequency bands (predominantly the alpha band)^31,50,61^. Therefore, the selective involvement of the beta frequency band during the menstrual cycle should not be over interpreted, as more studies are needed to corroborate this finding.

In conclusion, this MEG study directly addresses the dynamic changes in the brain activities across the MC. We showed that brain dynamics change during the MC and, in particular, we observed more flexibility during the peri-ovulatory phase. Furthermore, we found increased recruitment of areas belonging to the limbic system during the peri-ovulatory phase.

This paper has several limitations. The scalp electromyogram was not recorded to remove the low-amplitude, low-frequency electromyogram artefacts that characterise the psycho-emotional state and could introduce biases. Furthermore, we could not find any correlation between the functional repertoire and the blood levels of sex hormones. Further MEG studies are needed to elucidate the biological mechanisms that underpin the observed changes in the brain dynamics across the MC as a function of physiological factors, such as sex hormones.

Moreover, although brain flexibility can be estimated in different ways^62^, in the present study, we defined brain flexibility through the construct of neuronal avalanches and, in particular, via the functional repertoire. With respect to the other metrics, the functional repertoire aims at capturing the brain dynamics at the large scale, in a mathematical principled way that is solidly established in the context of statistical mechanics. That is, each functional pattern is defined across the whole brain and, as such, the status of each and every region equally determines the identity of a brain state. Our study might have positive implications for clinical medicine provided that neurological diseases, such as migraine or epilepsy, are affected by the MC. Neurologists and gynaecologists might find investigations of brain dynamics in women useful for women with neurological disorders, perhaps leading to personalized risk assessment for neurological symptoms.

### Supplementary Information

Supplementary Information.

## Data Availability

The data that support the findings of this study are available from the corresponding author, PS, upon reasonable request.
